# The CD44s Isoform is a Potential Biomarker for Predicting Craniopharyngioma Recurrence in Children

**DOI:** 10.1007/s12017-024-08797-y

**Published:** 2024-07-17

**Authors:** K. Bajdak-Rusinek, N. Diak, E. Gutmajster, A. Fus-Kujawa, M. Ciupińska, B. Kalina-Faska, A. Trybus, M. Grajek, M. Kalina, M. Mandera

**Affiliations:** 1 Department of Medical Genetics, Faculty of Medical Sciences in Katowice, Medical University of Silesia, Medykow 18 Street, 40-752 Katowice, Poland; 2 Biotechnology Centre, Silesian University of Technology, Gliwice, Poland; 3 Department of Pathomorphology and Molecular Diagnostics, Medical University of Silesia, 40-752 Katowice, Poland; 4 Department of Pediatrics and Pediatric Endocrinology, Faculty of Medical Science, Medical University of Silesia, Katowice, Poland; 5 Students Scientific Society, Faculty of Medical Sciences in Katowice, Medical University of Silesia, Katowice, Poland; 6 Center for Cardiovascular Research and Development, American Heart of Poland, 40-028 Katowice, Poland; 7 Department of Pediatric Neurosurgery, Medical University of Silesia, Katowice, Poland

**Keywords:** Craniopharyngioma, Children, Tumor recurrence, CD44

## Abstract

Adamantinomatous craniopharyngioma (ACP) is an intracranial tumor considered partly malignant due to its ability to infiltrate surrounding structures and tendency to relapse despite radical resection. CD44 is a known stem cell marker in ACP and is upregulated in cell clusters of invasive ACP protrusions; however, the functions of its alternative splicing isoform variants, CD44s and CD44v1-10, have not yet been studied in terms of ACP recurrence, despite their confirmed roles in cancer development and progression. In this study, we first confirmed the difference in total CD44 expression between samples from patients who experienced relapse and those from patients who did not. Moreover, our findings showed that, in recurrent samples, the predominant isoform expressed was CD44s, which might indicate its significance in predicting ACP recurrence. The association between increased CD44 expression and recurrence may lead to the development of prognostic markers of ACP aggressiveness and relapse potential; however, further studies are needed to clarify the exact mechanism of CD44 expression.

## Introduction

Adamantinomatous craniopharyngiomas (ACPs) are uncommon, epithelial tumors of the sellar region that arise along the path of the craniopharyngeal duct. In the pediatric population, these tumors constitute approximately 5–15% of intracranial tumors (Gabay et al., 2023). Although the WHO classifies ACP as a benign tumor, it is clinically considered “partially malignant” because of its tendency to infiltrate surrounding structures and relapse (Louis et al., 2007).

The main therapeutic methods are surgery and radiotherapy, but these are not always curative and can often contribute to further damage. The most common complication of all forms of craniopharyngioma therapy, which has a strong and unfavorable impact on survival, is tumor recurrence. Despite the need for radical surgery, the recurrence rate is as high as 5–57% (Clark et al., 2015), and relapse risk factors are still unknown (Negoto et al., 2015).

The molecular etiology of ACP is a mutation in *CTNNB1* (encoding β-catenin), leading to excessive activation of the WNT pathway. Interestingly, b-catenin-accumulating cell clusters were demonstrated within the invasive tumor protrusions of adamantinomatous craniopharyngiomas (Campanini et al., 2022). Additionally, the stem cell marker CD44 is consistently and predominantly coexpressed in these cell clusters, suggesting that these cells are cancer stem cells that contribute to tumor recurrence (Wang et al., 2019).

**Fig. 1:**
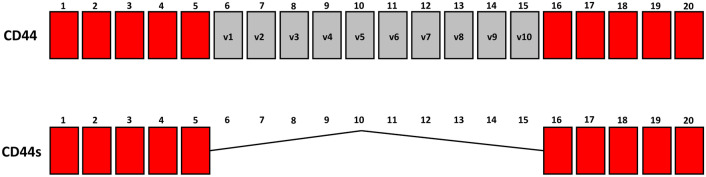
Structure of the CD44 gene. CD44s are encoded by exons (1 to 5) and (16 to 20). Exons 6–15 (v1 to v10) generate CD44 variants through alternative splicing

CD44 is a surface molecule that exists as a series of isoforms resulting from the alternative splicing of 10 “variant” exons (Fig. 1). Little is known about the expression and function of individual isoform variants. Extensive research has shown that both CD44s and CD44v play various important roles in cancer development and progression (Hassn Mesrati et al., 2021). However, the conflicting data presented demonstrate that further research is required to determine which isoform has a greater impact on key tumorigenic features and to define the underlying mechanisms by which these isoforms promote the tumorigenicity and aggressiveness of a specific tumor.

In our study, we analyzed material from patients who experienced ACP recurrence in the postoperative period (n = 10) and from recurrence-free patients (n = 25). We analyzed the overall expression level of CD44 as a stem cell determinant and then examined the expression levels of individual CD44 isoforms.

## Materials and Methods

### Patients and Tissue Specimens

Research was carried out on material obtained during the resection of craniopharyngiomas from pediatric patients between 2000 and 2019. Subsequently, the material was delivered to the Pathomorphology Department for histopathological analysis. The tissue was fixed with formalin and embedded in paraffin according to the manufacturer’s instructions. A WHO grade 1 diagnosis was confirmed for all patients. The remaining material posthistological analysis was deposited in the department’s archives. Fixed tissues from 35 craniopharyngioma patients were obtained from the archive, including 10 patients with recurrence.

Patients included in this study were treated exclusively by surgical resection. They did not receive other treatments such as radiotherapy or intracapsular interventions. The extent of resection depended on the tumor involvement of the hypothalamus, which was assessed before surgery in each patient according to the Puget scale (Puget et al., 2007). Total radical resection was performed only for Puget 0 or 1 tumors, and its effectiveness was assessed by intraoperative confirmation of complete resection and postoperative imaging without the presence of residual tumor. Recurrent ACP was defined by observation of tumor recurrence using magnetic resonance imaging. The mean age of the subjects was 10.7 years (2–17 years, SD = 4.06), and 54.3% were females and 45.7% were males.

### RNA Isolation

The formalin-fixed paraffin-embedded (FFPE) material was cut into 10 μm thick slices using a CUT 4050 microtome (microTec Laborgeräte GmbH, Walldorf, Germany). Isolation was performed immediately with a dedicated RNeasy® FFPE Isolation Kit (Qiagen, Hilden, Germany) according to the manufacturer’s protocol. Deparaffinizing solution (Qiagen, Hilden, Germany) and DNAse (Qiagen, Hilden, Germany) were used when necessary. Incubations were carried out on a TS-100 thermomixer (BioSan, Józefów, Poland). The quality and quantity of the isolated material were assessed using a Nanodrop spectrophotometer (Thermo Fisher Scientific, Waltham, MA, USA). The isolated RNA was kept at − 80 °C until further analyses.

### Real-Time Quantitative Polymerase Chain Reaction

RNA was reverse-transcribed into cDNA one week after isolation using the Transcriptor First Strand cDNA Synthesis Kit (Roche, Mannheim, Germany) according to the supplier’s protocol. The samples were incubated in a Verity thermocycler (Applied Biosystems, Darmstadt, Germany). Relative expression levels were measured in triplicate in a Roche Light Cycler 480 using Power SYBR Green PCR Master Mix (Applied Biosystems, Darmstadt, Germany), 300 mM primers (Table 1), and 1/15 cDNA stock. Relative expression levels were calculated and normalized to GAPDH levels using the Pfaffl method (Pfaffl, 2001).

**Table 1:** Primers used in qRT‒PCR analysis

Gene	Forward primer (5′–3′)	Reverse primer (5′–3′)
GAPDH	GCACCGTCAAGGCTGAGAAC	ATGGTGGTGAAGACGCCAGT
CD44_total	ATAATTGCCGCTTTGCAGGTGTATT	ATAATGGCAAGGTGCTATTGAAAGCCT
CD44s	ATAATAAAGGAGCAGCACTTCAGGA	ATAATTGTGTCTTGGTCTCTGGTAGC
CD44v2	ATAATCAGCAACTGAGACAGCAACCAA	ATAATAACCAATCCCAGGTTTCTTGCC
CD44v3	ATAATGGCTGGGAGCCAAATGAAGAAA	ATAATCATCATCATCAATGCCTGATCCAGA
CD44v4	ATAATCAGTGGAACCCAAGCCATTCAA	ATAATCCTTGTGGTTGTCTGAAGTAGCAC
CD44v5	ATAATGAAACTGGAACCCAGAAGCACA	ATAATTGATGCTCATGGTGAATGAGGG
CD44v6	ATAATCAGAAGGAACAGTGGTTTGGCA	ATAATGTCTTCTTTGGGTGTTTGGCGA
CD44v7	ATAATTGCAAGGAAGGACAACACCAAG	ATAATGGGTGTGAGATTGGGTTGAAGA
CD44v8	ATAATACGCTTCAGCCTACTGCAAA	ATAATAAGAGGTCCTGTCCTGTCCAAA
CD44v9	ATAATGAGCTTCTCTACATCACATGAAGGC	ATAATGTCAGAGTAGAAGTTGTTGGATGGTC
CD44v10	ATAATACCTCTCATTACCCACACACGA	ATAATTAGCTGAGGTCACTGGGATGAA

### Statistical Analysis

The data distribution was checked with the Shapiro‒Wilk test, and differences between two groups were tested with the Mann‒Whitney test. The analyses were performed with Statistica v.13.3 (StatSoft, TIBO, Naperville, IL, USA) and the online www.GIGAcalculator.com for the recurrence risk assessment. The chosen significance level was p < 0.05. Statistical analyses of the qRT‒PCR data were performed with Microsoft Excel software. Normalized relative expression levels were used to calculate the mean and the SD of all the experiments.

## Results

### Pattern of Recurrence

**Fig. 2:**
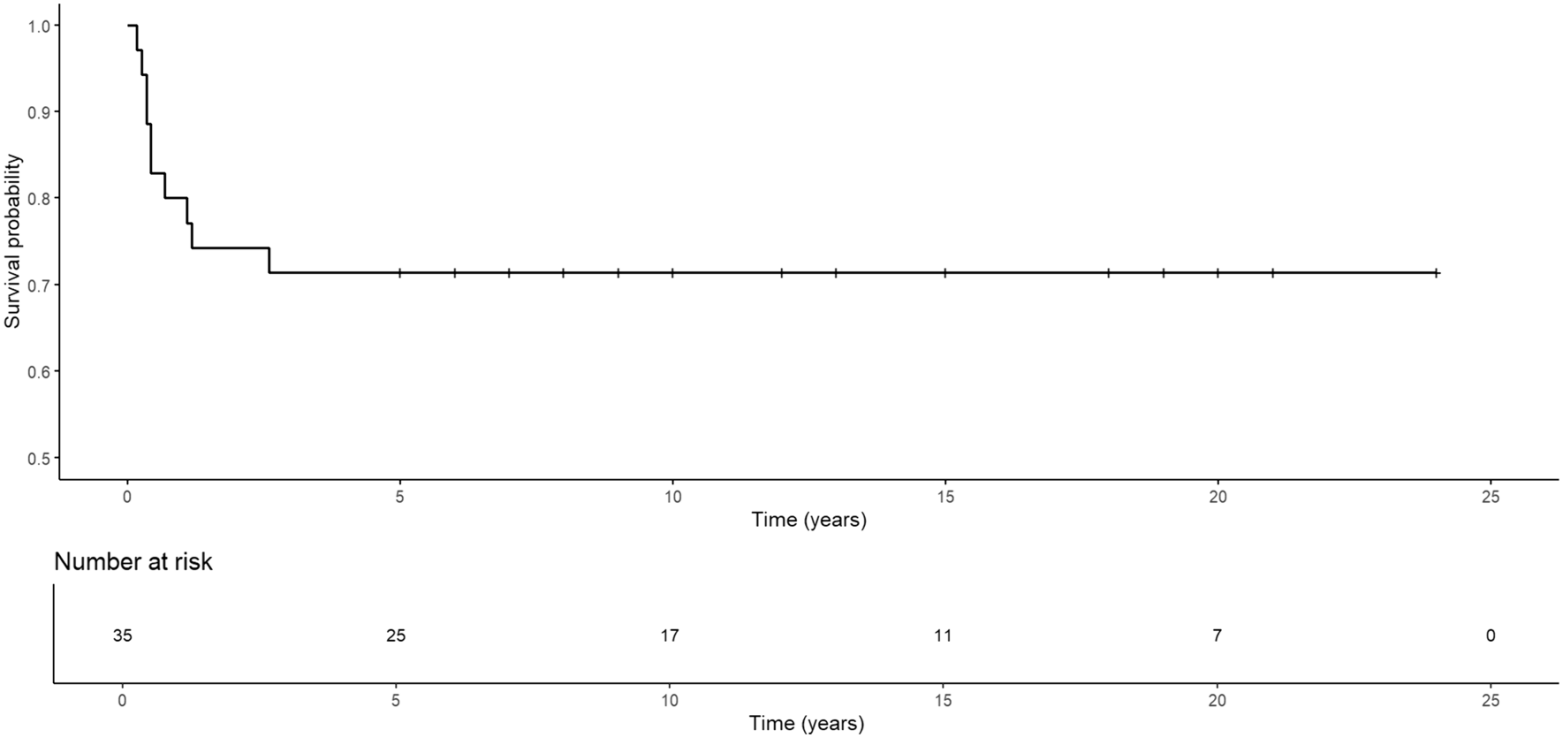
Recurrence-free survival curves for patients with craniopharyngioma after radical excision based on Kaplan‒Meier curve analysis

Thirty-five children with ACP were included in this study—16 boys (45.7%) and 19 girls (54.3%) (mean age 10.7 years; range 2–17). Of these children, 10 had tumor recurrence (5 boys and 5 girls) within 31 months (mean 8.9 months) after initial surgery. The overall 5-year recurrence-free survival rate was 71.4% (Fig. 2).

### CD44 as a Marker of Poor Prognosis and Clinical Recurrence in Patients with Craniopharyngioma

**Fig. 3:**
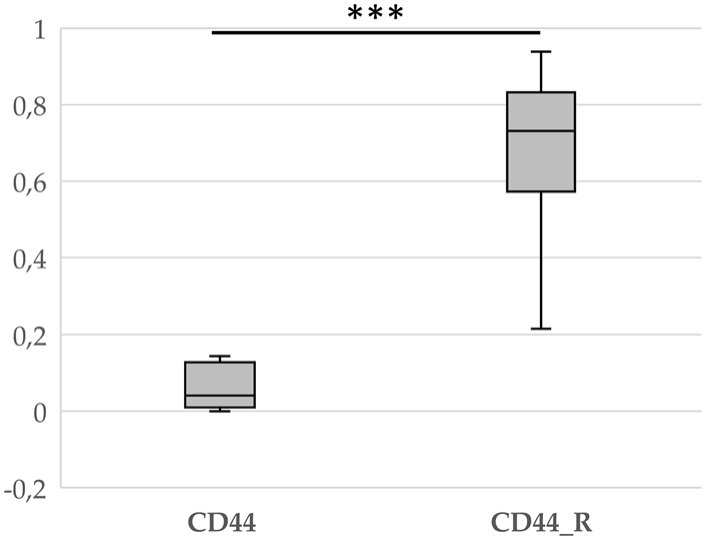
Box plot showing the fold change in CD44 gene expression in patients with (CD44_R) and without (CD44) craniopharyngioma recurrence. ***p < 0.001

CD44 expression appears to be a marker of poor prognosis and clinical recurrence in many types of cancer, such as kidney, thyroid and colorectal cancer (Hassn Mesrati et al., 2021). CD44 is also a known stem cell marker in craniopharyngiomas. Therefore, we first checked whether there was a difference in CD44 expression between samples obtained from patients without recurrence and samples obtained from patients who developed recurrence. The results clearly showed differences between these two groups (Fig. 3).

### The CD44s Isoform as a Predictor of Craniopharyngioma Recurrence

The next stage involved checking the profiles of individual isoforms in samples taken from patients who experienced recurrence and from those who did not.

**Fig. 4:**
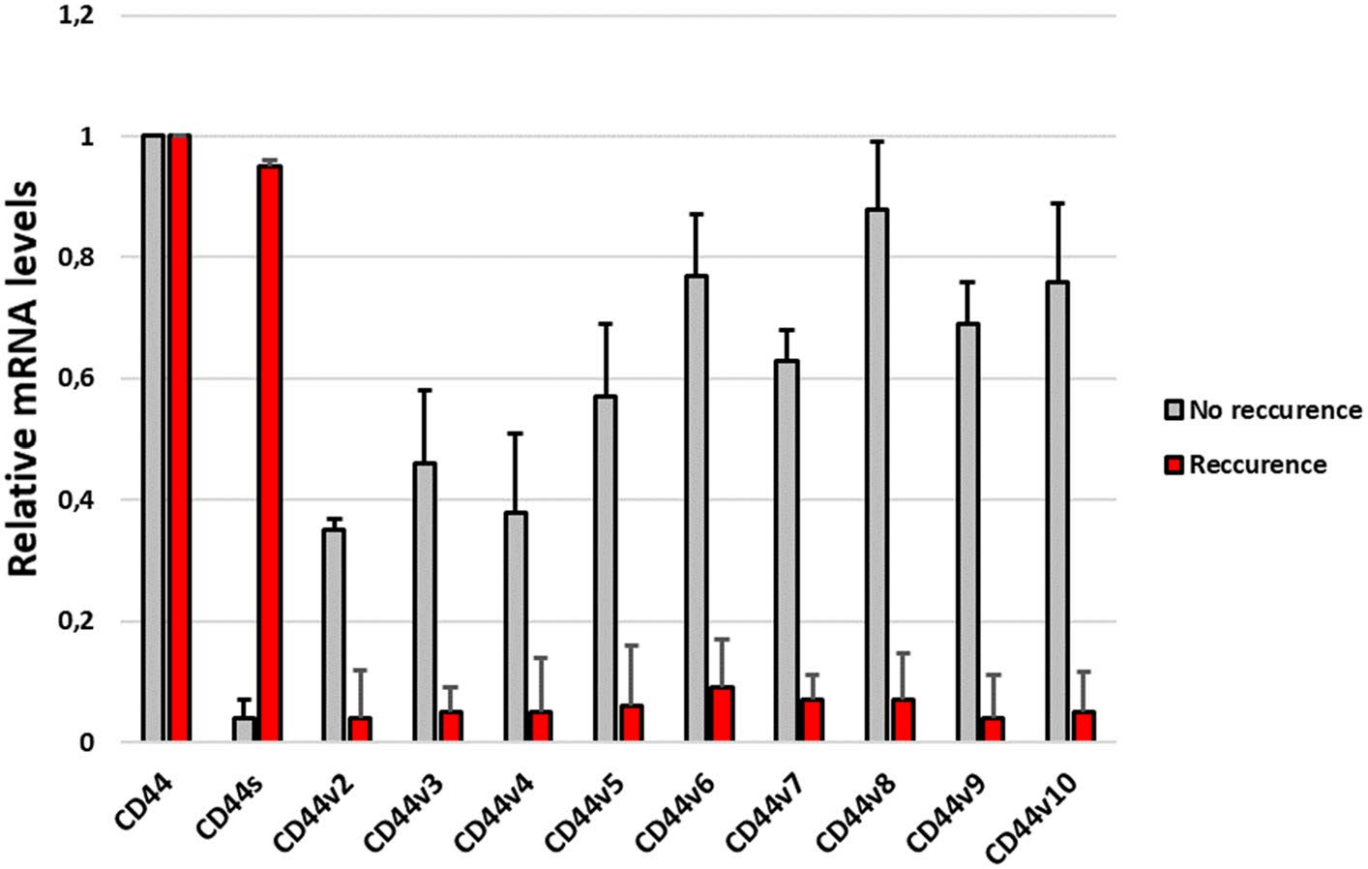
mRNA expression levels of CD44 isoforms in patients with and without craniopharyngioma recurrence

Our results showed that in patients with recurrence, the predominant isoform expressed was CD44s. In turn, in patients without recurrence, CD44v2-v10 expression predominated, while CD44s were practically absent (Fig. 4).

**Fig. 5:**
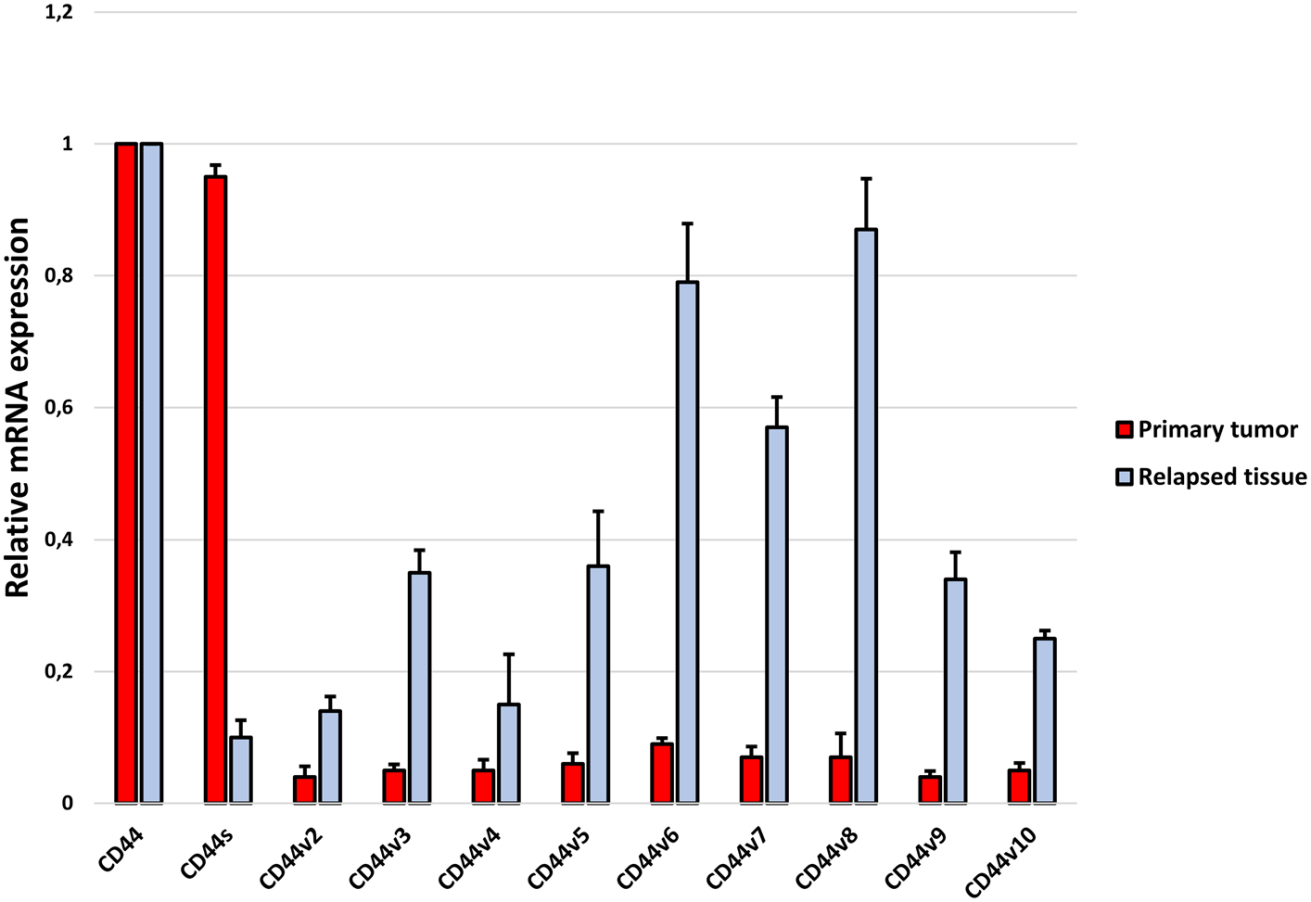
mRNA expression levels of CD44 isoforms in tissue from the primary tumor of patients who experienced recurrence and the relapsed tissue itself

Finally, we checked the distribution of individual isoforms in the tissue of the recurrence itself and compared it with the primary tumor tissue of patients who experienced recurrence (Fig. 5). The results showed that, as in the tissues of non-relapsed patients, the predominant isoforms in the relapse tissue itself are CD44v2-v10, while CD44s is expressed at a very low levels. These may indicate a different role of individual isoforms in the process of recurrence of craniopharyngioma in children.

Overall, our findings may constitute a basis for assessing the molecular profile of craniopharyngiomas and constitute a prognostic marker for potential tumor recurrence.

## Discussion

The recurrence rate of craniopharyngiomas ranges from 5 to 57% (Clark et al., 2015). Numerous studies indicate that the critical moment for tumor recurrence is the first 5 years after surgery (Negoto et al., 2015). Among our patients, the mean time to recurrence was approximately 8.9 months, and all recurrences occurred within 3 years. The 5-year recurrence-free survival rate of more than 70% in our patients (Fig. 2) was similar to the recurrence-free survival rate of 74–81% reported in other pediatric series of craniopharyngiomas after resection (Duff et al., 2000; Effenterre & Boch, 2002).

Regarding the molecular causes of tumor recurrence, studies have shown that the cause is the accumulation of β-catenin within invasive ACP protrusions. Interestingly, these cells also coexpress the stem cell marker CD44 (Wang et al., 2019). Several studies have indicated that the upregulation of CD44 in many cancer types, including prostate, ovarian, and brain cancer, is correlated with aggressive biological behavior and poor prognosis (Hassn Mesrati et al., 2021). In turn, a clinical study of patients with hepatocellular carcinoma showed that increased expression of the CD44 marker in the perioperative period may predict early relapse after radical surgery (Choi et al., 2015). This was confirmed in our study, where we observed a significant difference in CD44 expression between patients who experienced recurrence and those who did not (Fig. 3).

CD44 is a cell surface glycoprotein implicated in cell adhesion, proliferation and metastatic tumor growth. The gene is composed of two sets of exons, namely, standard (CD44s) and variable (CD44v) exons (Fig. 1), but the functions of the individual isoform variants are still unclear (Hassn Mesrati et al., 2021). Extensive evidence indicates that both the CD44s and CD44v isoforms are overexpressed in a variety of cancers and play key roles in their development. However, their clinical significance is still under debate due to conflicting findings regarding their prognostic and predictive value.

A study by Gotod et al. showed that the expression of the isoforms CD44v6 and CD44v2 was a useful predictor of poor outcome in patients after resection of pancreatic cancer (Gotoda et al., 1998). In turn, Li et al., using an antibody against CD44s, reported reduced growth, metastasis, and postradiation recurrence of pancreatic xenograft tumors in mice (Li et al., 2014). However, our results indicate that the dominant isoform expressed in patients with ACP who developed recurrence is CD44s (Fig. 4). Interestingly, in tissue collected from the recurrence itself, the CD44s isoform is the least expressed of all the others (Fig. 5). Moreover, the expression profile of individual isoforms from the relapsed tissue is analogous to the profile observed in patients without relapse. This may mean that the CD44s isoform is only needed to initiate disease relapse, but further, more detailed analyses are needed to confirm this.

In conclusion, in our current work, we observed for the first time that increased expression of CD44, specifically the CD44s isoform, is associated with the recurrence of craniopharyngioma. Nevertheless, further large-scale studies are needed to confirm our results, as are additional preclinical studies to elucidate the exact mechanism of CD44 expression in children with craniopharyngioma who develop recurrence after radical surgical resection. Determining the expression of individual CD44 isoforms may be a useful prognostic marker in the assessment of craniopharyngioma recurrence in children.

## Data Availability

The datasets used and analyzed in the current study are available from the corresponding author on reasonable request.
